# The distribution of esophageal cancer patients enrolled in care at the Uganda Cancer Institute by sub-regions, districts and ethnicity

**DOI:** 10.4314/ahs.v24i1.24

**Published:** 2024-03

**Authors:** Siraji Obayo, Yusuf Mulumba, Cheryl L Thompson, Michael K Gibson, Matthew M Cooney, Jackson Orem

**Affiliations:** 1 Uganda Cancer Institute; 2 Case Western Reserve University, Case Comprehensive Cancer Centre; 3 Vanderbilt University Medical Centre, Vanderbilt-Ingram Cancer Center

**Keywords:** Esophageal cancer, sub-regions, districts, ethnicity, Uganda

## Abstract

**Background:**

There is limited published data regarding the distribution of esophageal cancer patients by sub-regions, districts and ethnicity in Uganda.

**Objectives:**

To study the distribution by sub-regions, districts, ethnicity and sub-regions post-care outcomes of esophageal cancer patients in care over ten years at the Uganda Cancer Institute.

**Methods:**

Patients' charts with confirmed diagnoses of esophageal cancer for 2009–2019 were identified. Case information, which included demographics, clinical presentation, distribution by sub-regions, districts, ethnicity and sub-regions post-care outcomes, were retrospectively abstracted.

**Results:**

Central 671(34.15%), Southwestern 308(15.67%), Elgon 176(8.95%) and East central 163(8.29%) sub-regions had most patients. Mostly from administrative districts of Wakiso 167(8.50%), Mbarara 51(2.59%), Tororo 53(2.70%), Busia 33(1.68). Baganda, Banyakole, Bagisu and Basoga ethnic groups predominate. Patients from neighbouring countries were mainly from Rwanda 56(2.85%), South Sudan 24(1.22%), then Kenya 21(1.07%), and Rwandese, Dinka and Luo by ethnicity, respectively. Central and Southwestern sub-regions had the most post-care outcomes of the patients regarding living, death, and loss to follow-up.

**Conclusion:**

Patients are commonly from the administrative districts of Central, Southwestern, Elgon and East Central sub-regions and neighbouring countries of Rwanda, South Sudan and Kenya. Baganda, Banyakole, Bagisu and Basoga are the main ethnic groups. Central and Southwestern sub-regions are with most post-care outcomes.

## Introduction

Esophageal cancer is the seventh most common cancer and the sixth leading cause of cancer death worldwide^1^. More than 80% of cases and deaths from esophageal cancer occur in developing countries 2-4. The incidence of esophageal cancer varies globally, with a higher incidence in areas such as Eastern Asia, South Central Asia, South Africa, Eastern Africa, and Northern Europe 1-4. In Uganda, one of the countries comprising the East African sub-region, the incidence and trend of esophageal cancer is increasing 5-9. In Uganda, esophageal cancer ranks sixth and is the third common cause of cancer-related death, accounting for 8.7%^9^. The male-to-female risk ratio is about 2:1 6, 9, 10. Despite the rising esophageal cancer incidence in Uganda, there is limited data about the clinical characteristics, distribution by sub-regions, districts, ethnicity and sub-regions post-care outcomes for this tumor type. This study aimed to characterize esophageal cancer patients seeking care over ten years in terms of distribution by sub-regions, districts, ethnic groups and sub-regions post-care outcomes. Therefore, the baseline data obtained from this study will be the first important step for developing resources, enhancing esophageal cancer care in our population, and performing additional research.

## Methods

This was a retrospective chart review study of confirmed esophageal cancer patients referred to the Uganda Cancer Institute, a national referral cancer center, between 2009 and 2019 for care. Data collected on each patient's chart included age, sex, occupation, Body Mass Index (BMI), Eastern Cooperative Oncology Group (ECOG) performance status, main referral complaint, diagnostic method, ethnicity, their districts, sub-region locations, and sub-regions post-care outcomes. Data were collected and stored using the RedCAP database. This study was approved by the Ugandan National Council for Science and Technology and the Uganda Cancer Institute.

### Statistical analysis

Mean values and standard deviations were calculated for continuous variables. Counts of categorical variables described the distributions of demographic, clinical characteristics variables and esophageal cancer in each sub-region, district, or from a neighbouring country. The relationship between patient's sub-regions by districts, ethnicity and post-care outcomes was determined by cross-tabulation.

## Results

Out of 1965 patients whose charts were reviewed, 1380(70.23 %) were males, and 585(29.77 %) were females with a mean age of 60.20 years (SD12.66). Their average BMI was 17.61 (SD 3.16). Most of the patients, 1475(75.06%), were in agriculture, followed by professionals, 490(24.94%).

Patients commonly presented with progressive dysphagia 1577(80.30%) as a main complaint, followed by epigastric pain 260(13.20%), then odynophagia 128(6.50). Their diagnostic methods were as follows, upper gastrointestinal endoscopy plus biopsy in 1472(74.91%), then barium swallow followed by upper gastrointestinal endoscopy plus biopsy in 493(25.09%) patients. Most patients were under weight at presentation, 1224(62.29%), and the majority had the ECOG performance status of 1-3, 1813(92.26%), Table 1.

**Table 1:** Demographic and clinical characteristics of esophageal cancer patients

Characteristic	Mean (SD)	Numbers	Proportion (%)
**Sex**			
Male		1380	70.23
Female		585	29.77
**Age**	60.20 (12.66).		
≤ 40		117	5.95
41-50		347	17.66
51-60		618	31.45
≥ 61		883	44.94
**Occupation**			
Agriculture		1475	75.06
Professionals		490	24.94
**Main compliant**			
Dysphagia		1577	80.30
Epigastric pains		260	13.20
Odynophagia		128	6.50
**Diagnostic methods**			
Upper endoscopy plus biopsy		1472	74.91
Barium swallow then Upper endoscopy plus biopsy		493	25.09
**Body Mass Index (BMI, Kg/m^2^)**	17.61(3.16).		
Underweight (<18.5)		1224	62.29
Normal weight (18.5-24.9)		715	36.39
Overweight (25.0-29.9)		26	1.32
**ECOG/Performance status**			
0		44	2.24
1		562	28.60
2		730	37.15
3		521	26.51
4		108	5.50

SD=Standard deviation

**Figure 1:**
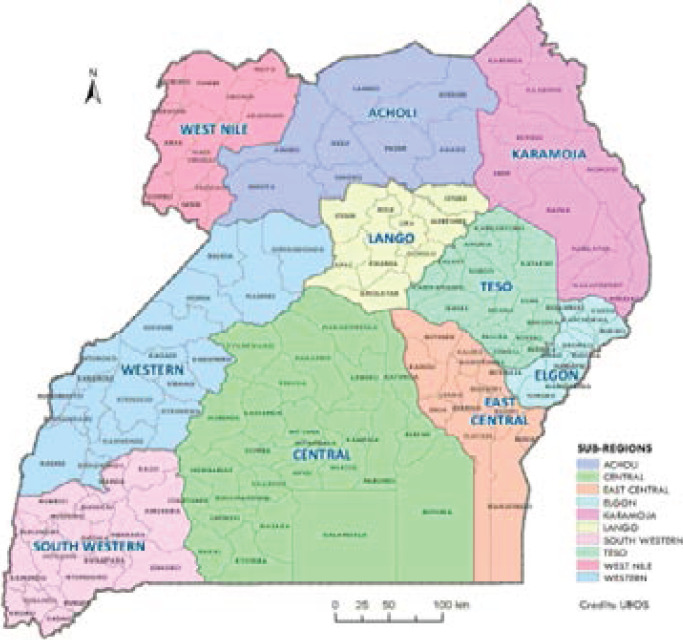
Map of Uganda showing sub-regions and corresponding districts

Distribution of these patients by sub-region, district, and ethnicity, most of them were from the Central sub-region, 671(34.15%), Table 2, mainly from the districts of Wakiso 167(8.50%), Kampala 145(7.38%), Mukono 57(2.90%) and Masaka 44(2.24%), Figure 1, Table 3, mostly Baganda 662(33.69) and Baruli 6(0.31) by ethnic group, Table 5. Followed by Southwestern sub-region 308(15.67%), Table 2, mainly from the districts of Mbarara 51(2.59%), Ntungamo 36(1.83%), Bushenyi 33(1.68%) and Kabale 31(1.58%), Figure 1, Table 3, mainly Banyakole 228(11.60%) followed Bakiiga 54(2.75%) by ethnicity, Table 5.

**Table 2:** Esophageal cancer patients who got care by sub-region and neighbouring countries

Sub-Region	N (%)	Neighbouring countries	N (%)
Central	671(34.15)	Rwanda	56(2.85)
Southwestern	308(15.67)	South Sudan	24(1.22)
Elgon	176(8.95)	Kenya	21(1.07)
East Central	163(8.29)	Burundi	6(0.31)
Western	145(7.38)	Congo	2(0.10)
Teso	139(7.07)	Somalia	1(0.05)
West Nile	107(5.45)		
Lango	87(4.43)		
Acholi	51(2.60)		
Karamoja	8(0.41)		
**Total**	**1855(94.40)**		**110(5.60)**

**Table 3:** Distribution in Central, Southwestern, Elgon, East Central and Western districts

	Central	Sub-region N (%)				East Central			Western

		Southwestern		Elgon					
**District N (%)**									
Wakiso	167(8.50)	Mbarara	51(2.59)	Tororo	53(2.70)	Busia	33(1.68)	Kabarole	41(2.10)
Kampala	145(7.38)	Ntungamo	36(1.83)	Mbale	34(1.73)	Jinja	32(1.63)	Hoima	23(1.17)
Mukono	57(2.90)	Bushenyi	33(1.68)	Sironko	24(1.22)	Iganga	29(1.47)	Kyenjojo	21(1.07)
Masaka	44(2.24)	Kabale	31(1.58)	Manafwa	20(1.02)	Kamuli	19(0.96)	Kibaale	15(0.76)
Rakai	29(1.48)	Rukungiri	31(1.58)	Bukwo	16(0.81)	Butaleja	15(0.76)	Masindi	14(0.71)
Luwero	27(1.37)	Isingiro	23(1.17)	Bulambuli	9(0.46)	Kaliro	7(0.36)	Kyegegwa	9(0.46)
Mpigi	27(1.37)	Sheema	21(1.07)	Bududa	7(0.36)	Bugiri	6(0.31)	Kamwenge	6(0.31)
Buikwe	24(1.22)	Kisoro	19(0.97)	Kapchorwa	7(0.36)	Luuka	6(0.31)	Kasese	5(0.25)
Mityana	20(1.02)	Kiruhura	17(0.87)	Kween	5(0.25)	Mayuge	6(0.31)	Kiryandongo	4(0.20)
Kalungu	17(0.87)	Mitooma	14(0.71)	Namisindwa	1(0.05)	Namutumba	5(0.25)	Kakumiro	3(0.15)
Mubende	17(0.87)	Ibanda	13(0.66)			Namayingo	3(0.15)	Kagadi	2(0.10)
Nakaseke	15(0.76)	Kanungu	9(0.46)			Buyende	2(0.10)	Budibugyo	1(0.05)
Butambala	12(0.61)	Buhweju	4(0.20)					Ntoroko	1(0.05)
Kayunga	12(0.61)	Rubirizi	4(0.20)						
Ssembabule	11(0.56)	Rubanda	2(0.10)						
Kiboga	10(0.51)								
**Total**	**634(32.27)**	**308(15.6.7)**		**176(8.96)**		**163(8.29)**		**145(7.38)**	

Elgon sub-region was at 176(8.95%), Table 2, mainly from the districts of Tororo 53(2.70%), Mbale 34(1.73%), Sironko 24(1.22%) and Manafwa 20(1.02%), Figure 1, Table 3. The most common ethnic groups were Bagisu 94(4.78%), Japadhola 30(1.53%) and Sabiny 28(1.42%), Table 5.

East Central sub-region was at 163(8.29%) Table 2, mainly from Busia 33(1.68%), Jinja 32 (1.63%), Iganga 29(1.47%) and Kamuli 19(0.96%) districts, Figure 1, Table 3. Mostly Basoga 110(5.60%), Basamia 36(1.83%) and Banyole 15(0.76%) by ethnicity, Table 5. From Western sub-region 145(7.38%) Table 2, Kabarole district contributed 41(2.10%) followed by Hoima 23(1.17%), Kyenjojo 21(1.07%) then Kibaale 15(0.76%), Figure 1, Table 3. The ethnic groups were mainly Batooro 67(3.41%) and Bunyoro 48(2.44%) with Banyakole contributing 13(0.66%) and Bakiiga 8(0.41%) in this sub-region, Table 5.

Other districts from the Central sub-region included Bukomansimbi, Gomba, Kyotera, Lwengo and Nakasongola, which accounted for 30(1.53%), with each district having six esophageal cancer patients, Lyantonde 4(0.20%) and Kyankwanzi 3(0.15%).

Teso 139 (7.07%) was the sixth sub-region in esophageal cancer distribution and mainly from the districts of Kumi 27(1.37%), Soroti 25 (1.27%), Pallisa 16(0.81%) and Katakwi 15(0.76%), Figure 1, Table 4. They were mainly Etesot 112(5.69%) and Bagwere 17(0.87%) by ethnicity, Table 5. In West Nile sub- region 107(5.45%), Table 2, majority were from Arua district 48(2.44%) then Nebbi 17(0.87%), Koboko 13(0.66%) and Adjumani 9(0.46%), Figure 1, Table 4. The main ethnic groups were Lugbara 52(2.65%), Alur 26(1.33%) and Kakwa 14(0.71%), Table 5. Other patients were from neighbouring countries accounting for 110(5.60%), Table 2

**Table 4:** Distribution in Teso, West Nile, Lango, Acholi and Karamoja districts

	Teso	West Nile		Sub-region N (%)		Acholi		Karamoja	

				Lango					
**District N (%)**									
Kumi	27(1.37)	Arua	48(2.44)	Lira	40(2.04)	Gulu	23(1.17)	Abim	3(0.15)
Soroti	25(1.27)	Nebbi	17(0.87)	Oyam	15(0.76)	Kitgum	14(0.71)	Nakapiripirit	2(0.10)
Pallisa	16(0.81)	Koboko	13(0.66)	Dokolo	7(0.36)	Agago	4(0.20)	Amudat	1(0.05)
Katakwi	15(0.76)	Adjumani	9(0.46)	Kole	7(0.36)	Amuru	4(0.20)	Kotido	1(0.05)
Serere	11(0.56)	Maracha	6(0.31)	Apac	6(0.31)	Lamwo	3(0.15)	Moroto	1(0.05)
Amuria	10(0.51)	Yumbe	6(0.31)	Alebtong	5(0.25)	Pader	2(0.10)		
Bukedea	10(0.51)	Zombo	5(0.25)	Amolatar	4(0.20)	Nwoya	1(0.05)		
Kaberamaido	10(0.51)	Pakwach	2(0.10)	Otuke	2(0.10)				
Kibuku	7(0.36)	Moyo	1(0.05)	Kwania	1(0.05)				
Ngora	5(0.26)								
Budaka	3(0.15)								
**Total**	**139(7.07)**		**107(5.45)**		**87(4.43)**		**51(2.60)**		**8(0.40)**

**Table 5:** Ethnic groups in care that had the most esophageal cancer in each sub-region

						Ethnicity N (%)					
		
		First		Second		Third		Fourth		Fifth	
Sub-region	N (%)										
Central	671(34.15)	Ganda	662(33.69)	Ruli	6 (0.31)	Nubi	2(0.10)	Nkole	1(0.05)		
Southwestern	308(15.67)	Nkole	228(11.60)	Kiiga	54(2.75)	Fumbira	20(1.02)	Hororo	5(0.25)	Ganda	1((0.05)
Elgon	176(8.95)	Gishu	94(4.78)	Adhola	30(1.53)	Sabiny	28(1.42)	Etesot	22(1.12)	Nyole	2(0.10)
East Central	163(8.29)	Soga	110(5.60)	Samia	36(1.83)	Nyole	15(0.76)	Ganda	1 (0.05)	Gwere	1(0.05)
**Midwestern**	145(7.38)	Tooro	67(3.41)	Nyoro	48(2.44)	Nkole	13(0.66)	Kiiga	8(0.41)	Konjo	5(0.26)
Teso	139(7.07)	Etesot	112(5.69)	Gwere	17(0.87)	Kumam	10(0.51)				
West Nile	107(5.45)	Lugbara	52(2.65)	Alur	26(1.33)	Kakwa	14(0.71)	Madi 1	1(0.56)	Aringa	2(0.10)
Lango	87(4.43)	Lango	87(4.43)								
Acholi	51(2.60)	Acholi	51(2.60)								
Karamoja	8(0.41)	Kjong	6(0.31)	Pokot	1(0.05)	Ethur	1(0.05)				

In Lango sub-region 87(4.43%) Table 2, Lira district was at 40(2.04%), Oyam 15(0.76%), Dokolo and Kole at 7(0.36%) each, Apac 6(0.31%), Figure 1, Table 4, the patients were Lango 87(4.43%) by ethnic group, Table 5. Acholi sub-region 51(2.60%), Table 2, patients were mainly from the districts of Gulu 23(1.17%), Kitgum 14(0.71%), Agago and Amuru at 4(0.20%) each, Lamwo 3(0.15%) and they were Acholi 51(2.60%) by ethnic group, Table 4 and 5 respectively.

Karamoja sub-region was the list in esophageal cancer distribution, and patients were mainly from the districts of Abim 3(0.15%), Nakapiripirit 2(0.10%), Amudat, Kotido and Moroto each at 1(0.05%), Figure 1, Table 4. Karimojong were the majority 6(0.31%) by ethnicity, Table 5. Esophageal cancer patients from other countries, 110(5.60%), came in for care; the majority were from the neighbouring countries of Rwanda 56(2.85%), mostly Rwandese, followed by South Sudan 24(1.22%), mainly Dinka ethnic group, Kenya 21(1.07%) mostly Luo, Luya and Kalenjin by ethnicity, Burundi 6(0.31%) then Congo 2(0.10%), Table 2.

Other ethnic groups from the Mid-western sub-region included one each from Alur, Ganda, Ruli, and Wamba, accounting for 4(0.20%). Nubi 2(0.10%) in the West Nile sub-region.

Most living patients were from central 13(44.83%), followed by southwestern, East Central and Lango sub-regions, with each at 4(13.79%). Among the neighbouring countries, only Burundi had one patient still alive by the end of this study. Dead patients were mainly from central 394(34.17%), followed by Southwestern 165(14.31%), Elgon 107(9.28%), then East Central, 102(8.85%) sub-regions. Rwanda, as a neighbouring country, had the most patients, 32(2.78%), who sought care at the Uganda Cancer Institute dead, followed by South Sudan, 18(1.56%), then Kenya,8(0.69%). Lost to follow-up patients were mostly from Central 264(33.72%), Southwestern, 139(17.75%) then Elgon, 69(8.81%) sub-regions. Among the neighbouring countries, Rwanda, 24(3.07%), followed by Kenya, 13(1.66%), then South Sudan, 6(0.76%) had the most patients lost to follow-up, Table 6.

**Table 6:** Post-care outcomes of patients from each sub-region and neighbouring countries

Sub-region N (%)		Alive	Dead	LFU	Neighbouring countries N (%)		Alive	Dead	LFU
Central	671(34.15)	13(44.83)	394(34.17)	264(33.72)	Rwanda	56(2.85)	0(0.00)	32(2.78)	24(3.07)
South.W	308(15.67)	4(13.79)	165(14.31)	139(17.75)	South Sudan	24(1.22)	0(0.00)	18(1.56)	6(0.76)
Elgon	176(8.95)	0(0.00)	107(9.28)	69(8.81)	Kenya	21(1.07)	0(0.00)	8(0.69)	13(1.66)
East.C	163(8.29)	4(13.79)	102(8.85)	57(7.27)	Burundi	6(0.31)	1(3.45)	4(0.35)	1(0.13)
Western	145(7.38)	1(3.45)	81(7.03)	63(8.05)	Congo	2(0.10)	0(0.00)	2(0.17)	0(0.00)
Teso	139(7.07)	0(0.00)	80(6.93)	59(7.54)	Somalia	1(0.05)	0(0.00)	1(0.09)	0(0.00)
West.N	107(5.45)	1(3.45)	74(6.42)	32(4.09)					
Lango	87(4.43)	4(13.79)	47(4.08)	36(4.59)					
Acholi	51(2.60)	1(3.45)	31(2.68)	19(2.43)					
Karamoja	8(0.41)	0(0.00)	7(0.61)	1(0.13)					
Total	**1855(94.40)**	**28(96.55)**	**1088(94.36)**	**739(94.38)**	**110(5.60)**		**1(3.45)**	**65(5.64)**	**44(5.62)**

LFU = Lost to follow-up, South.W = Southwestern, East.C = East Central, West.N = West Nile.

## Discussion

One thousand nine hundred and sixty-five esophageal cancer cases were reviewed, and males were predominant with a male: female ratio of approximately 2:1. The male predominance demonstrated in this study is like other studies performed in Africa, particularly in the East African region 9-13. The male predominance could be explained by the fact that most of the known risk factors for esophageal cancer are related to behavior-smoking and excessive alcohol consumption, of which men are known to be worse consumers than women, as has been shown by studies in Africa and China 14-20.

Our study highlights esophageal cancer occurring in older age (>50 years), similar to earlier studies in the north rift valley of western Kenya, Tanzania, Mozambique, South Africa, Ethiopia, and Iran 11, 21-25. These findings can be explained by the fact that the chance of getting oesophageal cancer increases with age. However, the age may vary from country to country since it highly depends on the underlying population structure.

Most of the esophageal cancer patients seeking care were in agriculture, which is unsurprising as Uganda is an agricultural country with 72.1% of the working population employed in this sector. In our study, dysphagia was the most presenting symptom of esophageal cancer at the time of diagnosis. All the esophageal cancer was diagnosed through upper gastrointestinal endoscopy plus biopsy for histopathology, keeping with a comprehensive series of published reports in East Africa, South Central Asia and East Asia^13^, ^25^-^27^.

Our data demonstrate more than 60% of the patients were underweight at presentation. This result is explained by the fact that most of our patients (80.30%) had progressive difficulty swallowing, thus losing weight due to reduced intake and low body mass index before presenting for care. This finding is similar to the Zambian study that found 62.7% of esophageal cancer patients underweight 26.

In our study, most patients, 92.26%, had the Eastern Cooperative Oncology Group score (ECOG) performance status between 1-3. This finding contrasts with other studies from Korea, China, and France 28-31, where most patients had ECOG between 0-1. The high ECOG scores in our study could have probably indicated most of our patients being very sick, with the burden of preexisting disease hence a poor ECOG performance status.

Distribution, our study demonstrated most of the patients were from administrative districts of most sub-regions. This result is by the Tanzanian study for patients treated for esophageal cancer, which found higher incidence rates in administrative regions and the United States of America, where most patients were from metropolitan and urban areas 32, 33. Central had the most patients, 34.15% among the sub-regions; the high numbers from central might be related to more accessibility to care as our study hospital (Uganda Cancer Institute) is located in the central region urban area of Uganda.

It is worth noting that some sub-regions with high numbers of patients were significantly farther from the central sub-region and the Uganda Cancer Institute in our study. For example, Southwestern (15.67%), Elgon (8.95%) and East Central 8.29% sub-regions. An earlier study looking at risk factors for esophageal cancer among adults aged 40 years and above in eastern Uganda, Elgon sub-region, reported esophageal cancer as one of the biggest health problems and the leading cause of cancer-related mortality in the region 34. This report concurs with our findings explaining the burden of esophageal cancer in the Elgon sub-region districts despite not having cancer treatment hospitals. Most esophageal cancer patients accessing care from the neighbouring countries were from Rwanda, South Sudan, and Kenya.

Sub-regions post-care outcomes; our study highlights the Central and Southwestern sub-regions with the most post-care results of the patients regarding living, dead, and lost to follow-up. These post-care results could probably partly be explained by the many patients who came in for care from these sub-regions, among other contributing factors.

## Limitations

This study has some limitations. Only a tertiary national referral hospital was involved. Thus, cases referred from regional and district hospitals that didn't make it into care may have been missed; therefore, case ascertainment may be incomplete. We cannot exclude diagnostic bias based on interest, expertise, and access to diagnostic facilities.

## Conclusion

This study revealed higher proportions of esophageal cancer patients seeking care are mainly from administrative districts of Central, Southwest, Elgon, Eastern Central and Western sub-regions. Baganda, Banyakole, Bagisu, Basoga and Batooro are the main ethnic groups. Central and Southwestern sub-regions are with most postcare outcomes of the patients regarding living, death, and loss to follow-up. The neighbouring countries included patients from Rwanda, South Sudan, Kenya, then Congo. This study demonstrates the need for prioritizing esophageal cancer care to involve non-administrative rural areas of Uganda and nutritional assessment to help improve patients' nutritional status as they enrol in care, as most patients have low body mass index.

## References

[1] Sung H, Ferlay J, Siegel RL, Laversanne M, Soerjomataram I, Jemal A (2021). Global cancer statistics 2020: GLOBOCAN estimates of incidence and mortality worldwide for 36 cancers in 185 countries. CA Cancer J Clin.

[2] Ferlay J, Soerjomataram I, Dikshit R, Eser S, Mathers C, Rebelo M (2015). Cancer Incidence and Mortality Worldwide. Int. J. Cancer.

[3] Wong MCS, Hamilton W, Whiteman DC, Jiang JY, Qiao Y, Fung FDH (2018). Global Incidence and mortality of oesophageal cancer and their correlation with socioeconomic indicators temporal patterns and trends in 41 countries. Sci. Rep.

[4] Cheng ML, Zhang L, Borok M, Chokunonga E, Dzamamala C, Korir A (2015). The incidence of oesophageal cancer in eastern Africa: identification of a new geographic hot spot?. Cancer Epidemiol.

[5] Obayo S, Luswa L, Orem J, Ashley L F, Probert CS (2017). Gastrointestinal malignancies at five regional referral hospitals in Uganda. Afri Health Sci.

[6] Wabinga H, Nambooze S, Amulen P M (2013). Trends in the incidence of cancer in Kampala, Uganda 1991-2010. Int. J. Cancer.

[7] (2020). The global, regional, and national burden of oesophageal cancer and its attributable risk factors in 195 countries and territories, 1990-2017: a systematic analysis for the Global Burden of Disease Study 2017. Lancet Gastroenterol Hepatol.

[8] Huang J, Koulaouzidis A, Marlicz W, Lok V, Chu C, Ngai C H, Global Burden, Risk Factors, and Trends of Esophageal Cancer (2021). An Analysis of Cancer Registries from 48 Countries. An Analysis of Cancer Registries from 48 Countries. Cancers.

[9] Ferlay J, Ervik M, Lam F (2020). Global Cancer Observatory: Cancer Today. International Agency for Research on Cancer.

[10] Middleton DRS, Bouaoun L, Hanisch R, Bray F, Dzamalala C, Chasimpha S (2018). Esophageal cancer male to female incidence ratios in Africa: a systematic review and meta-analysis of geographic, time and age trends. Cancer Epidemiol.

[11] Wakhisi J, Patel K, Buziba N, Rotich J (2005). Esophageal cancer in north rift valley of western Kenya. African Health Sciences.

[12] Kachala R (2010). Systematic review: epidemiology of Oesophageal Cancer in Sub Saharan Africa. Malawi Medical Journal.

[13] Mabula D M, Rambau PF, Chalya PL, Hyasinta J, Mheta Koy, Mahalu W (2013). Endoscopic and clinicopathological patterns of esophageal cancer in Tanzania: experiences from two tertiary health institutions. World Journal of Surgical Oncology.

[14] Asombang AW, Chishinga N, Nkhoma A, Chipaila J, Nsokolo B, Manda-Mapalo M (2019). Systematic review and meta-analysis of esophageal cancer in Africa: epidemiology, risk factors, management and outcomes. World J Gastroenterol.

[15] Pampel F (2008). Tobacco use in sub-Sahara Africa estimates from the demographic health surveys, Soc. Sci. Med.

[16] Li Q, Hsia J, Yang G (2011). Prevalence of smoking in China in 2010. N Engl J Med.

[17] Millwood I Y (2013). Alcohol consumption in 0.5 million people from 10 diverse regions of China: prevalence, patterns and socio-demographic and health-related correlates. Int J Epidemiol.

[18] Liu S, Zhang M, Yang L, Li Y, Wang L, Huang Z (2017). Prevalence and patterns of tobacco smoking among Chinese adult men and women: findings of the 2010 national smoking survey. J Epidemiol Community Health.

[19] Yang X, Chen X, Zhuang M, Yuan Z, Nie S, Lu M (2017). Smoking and alcohol drinking in relation to the risk of esophageal squamous cell carcinoma: A population-based case-control study in China. Scientific Reports.

[20] Middleton D R S, Mmbaga B T, Menya D, Dzamalala C, Maro G N, Finch P (2022). Alcohol consumption and oesophageal squamous cell cancer risk in east Africa: finding from the large multicenter ESCCAPE case-control study in Kenya, Tanzania, and Malawi. Lancet Glob Health.

[21] Mmbaga E J, Deardorff K V, Mushi B, Mgisha W, Merritt M, Hiatt R A (2018). Characteristics of Esophageal Cancer Cases in Tanzania. J Glob Oncol.

[22] Come J, Castro C, Morais A, Cossa M, Modcoicar P, Tulsidas S (2018). Clinical and Pathologic Profiles of Esophageal Cancer in Mozambique: A Study of Consecutive Patients Admitted to Maputo Central Hospital. J Glob Oncol.

[23] Dandara C, Robertson B, Dzobo K, Moodley L, Parker M I (2016). Patient and tumour characteristics as prognostic markers for oesophageal cancer: A retrospective analysis of a cohort of patients at Groote Schuur Hospital. Eur J Cardiothorac Surg.

[24] Deybasso H A, Roba K T, Nega B, Belachew T (2021). Clinico-Pathological Findings and Spatial Distributions of Esophageal Cancer in Arsi Zone, Central Ethiopia. Cancer Management and Research.

[25] Aledavood A, Anvari K, Sabouri G (2011). Esophageal Cancer in Northeast of Iran. Iran J Cancer Prev.

[26] Asombang A W, Kasongo N, Muyutu J, Montiero J F G, Chishinga N, Chipaila J (2021). Descriptive analysis of esophageal cancer in Zambia using the cancer disease hospital database: young age, late stage at presentation. PAMJ.

[27] Jiang YX, Zhang DW, Chen Y, Sun HH, Xu SC, Gao HJ (2015). The characteristics of oesophageal squamous cell carcinoma: an analysis of 1317 cases in southeastern China. Contemp Oncol (Pozn).

[28] Jung HK, Tae CH, Lee HA, Lee H, Don Choi K, Park JC (2020). Treatment pattern and overall survival in esophageal cancer during a 13-year period: A nationwide cohort study of 6,354 Korean patients. PLoS ONE.

[29] Song T, Wan Q, Yu W, Li J, Lu S, Xie C, Wang H, Fang M (2017). Pretreatment nutritional risk scores and performance status are prognostic factors in esophageal cancer patients treated with definitive chemoradiotherapy. Oncotarget.

[30] Sun P, Zhang F, Chen C, An X, Li YH, Wang FH, Zhu ZH (2013). Comparison of the prognostic values of various nutritional parameters in patients with esophageal squamous cell carcinoma from Southern China. Journal of thoracic disease.

[31] Clavier JB, Antoni D, Atlani D, Ben AM, Schumacher C, Dufour P (2012). Baseline nutritional status is prognostic factor after definitive radiochemotherapy for esophageal cancer. Diseases of the Esophagus.

[32] (2016). Clinical and epidemiologic variations of esophageal cancer in Tanzania. World J Gastrointest Oncol.

[33] Kim S, DiPeri TP, Guan M, Placencio-Hickok VR, Kim H, Liu JY (2020). Impact of palliative therapies in metastatic esophageal cancer patients not receiving chemotherapy. World J Gastrointest Surg.

[34] Sabila N, Ndungutse D, Kiiza S, Ddamulira C (2019). Risk Factors for Esophageal Cancer among Adults Aged 40 Years and Above in Sebei Region, Eastern Uganda. Texila International Journal of Public Health.

